# Dog ectoparasites as sentinels for pathogenic Rickettsia and Bartonella in rural Guatemala

**DOI:** 10.21203/rs.3.rs-4656611/v1

**Published:** 2024-07-22

**Authors:** Yuexun Tian, Jose G. Juarez, Andrea M. Moller-Vasquez, María Granados-Presa, Francisco C. Ferreira, Pamela M. Pennington, Norma Padilla, Gabriel L. Hamer, Sarah A. Hamer

**Affiliations:** Texas A&M University; Universidad del Valle de Guatemala; Universidad del Valle de Guatemala; Universidad del Valle de Guatemala; Texas A&M University; Universidad del Valle de Guatemala; Universidad del Valle de Guatemala; Texas A&M University; Texas A&M University

**Keywords:** dog, ectoparasite, Guatemala, vector-bore, Rickettsia, Bartonella

## Abstract

**Background:**

Fleas and ticks serve as vectors of multiple pathogens in the genera *Rickettsia* and *Bartonellathat* cause diseases in humans and other animals. Although human rickettsiosis and bartonellosis have been reported in all countries in Central America, limited research has been conducted to investigate the natural cycles of flea- and tick-borne rickettsiosis and bartonellosis, especially in Guatemala.

**Methods:**

We evaluated dog parasites as sentinels for zoonotic disease risk in rural Guatemala by sampling ticks and fleas from dogs, which were then identified and individually screened for *Rickettsia* and *Bartonella*.

**Results:**

A total of 77 households were surveyed and 80.52% of them had dogs. Overall, 133 dogs were examined for fleas and ticks, of which 68.42% had fleas and 35.34% had ticks. A total of 433 fleas and 181 ticks were collected from the infested dogs, with an additional 33 ticks collected from house walls. Three flea species were identified: *Ctenocephalides felis* (70%), *Echidnophaga gallinacea*(11.8%), and *Pulex* sp. (17.8%). Among the collected ticks, 97% were *Rhipicephalus sanguineus* with the rest being *Amyblyomma cajennense, A. auricularium*, and *A. ovale*. *Rickettsia felis* were detected in six *C. felis*, in one *Pulex* sp., and in two *R. sanguineus*, while Candidatus *R. senegalensis* was detected in one *C. felis. Bartonella* was detected only in fleas, including three *Pulex*sp. infected with *B. vinsonii* subsp. *Berkhoffii, B. henselae*, and *Bartonella* sp., respectively, and 11 *C. felis* infected with *B. henselae*.

**Conclusions:**

This study reports Candidatus *R. senegalensis* and *B. vinsonii* subsp. *Berkhoffii*in Guatemala for the first time, and indicates the potential risk of human and dog exposure to *Rickettsia* and *Bartonella* species. These results show that dogs provide critical information relevant to managing human potential exposure to flea- and tick-borne pathogens in rural Guatemala.

## Background

Fleas and ticks are obligate blood-feeding ectoparasites of many animals including companion animals living in close contact with humans. Pathogens transmitted by these ectoparasites include bacteria of the genus *Rickettsia*, which are classified into two groups: the spotted fever group (e.g. *Rickettsia rickettsii, R. conorii, R. amblyommatis*), transmitted primarily by ticks; and the typhus group (e.g., *R typhi*), transmitted primarily by fleas and lice. *Rickettsia rickettsii* is the causative agent of Rocky Mountain spotted fever (RMSF), the most severe rickettsiosis (1), with *Amblyomma* spp. and *Rhipicephalus sanguineus* being the main vectors in the Americas (2). In the typhus group, the most common flea-borne species is *R. typhi*, causing murine typhus in humans, which is endemic to tropical and subtropical regions (Fang et al. 2017). Additionally, *R. felis* has emerged as a human pathogen with clinical presentations similar infections with flea-borne typhus (Fang et al. 2017). Fleas can also transmit *Bartonella* spp., including *B. henselae* and *B. clarridgeiae*, causative agents of cat scratch disease, with cats being the reservoir host and *Ctenocephalides felis* being the vector (3).

In Central America, human rickettsiosis has been reported in all countries. However, the annual number of rickettsiosis cases reported is low, which may be an underestimate due to the lack of efficient diagnoses (4) and surveillance. A recent systematic review reported that limited research has been conducted in Central America to study spotted fever group rickettsiosis, with the number of publications per country ranging from 1 in Guatemala to 27 in Costa Rica (5). The single publication in Guatemala reported an outbreak in 2007 where 10 out of 17 patients were confirmed or probable cases of spotted fever group rickettsiosis including two fatal cases (6). Prior research on *R. felis* in Guatemala includes the detection of *R. felis* in *C. felis* fleas (7) and one human case of *R. felis* infection (8). Flea-borne *Bartonella* infection has been reported in Guatemala with both *B. henselae* and *B. clarridgeiae* detected in cats and fleas (9). Therefore, there is a gap in understanding the prevalence of flea- and tick-borne rickettsiosis and bartonellosis in Guatemala. Further, given ~ 30% of dogs from across Guatemala were found to have heartworm infections (10), the use of heartworm prevention among dogs is likely low, and therefore the use of flea/tick ectoparasiticidal and anti-feeding products is also likely low. Because dogs frequent both outdoor and indoor environments, they can move vectors and pathogens across this interface and their infection status may provide an indication of local zoonotic disease risk to humans that share the household. This creates conditions for the establishment and expansion of such vectors intra- and peridomiciles, exposing humans to pathogens via contact with infective ticks and fleas.

With the increasing need for effective and efficient methods of emerging disease surveillance in the region our study aims to evaluate the potential of using dog ectoparasites as sentinels for zoonotic disease risk in a remote rural area of Guatemala. Our results can provide guidance to local public health authorities on the effectiveness of monitoring dogs for diseases that impact human health.

## Materials and Methods

### Ethic statement

The study was reviewed by the Research Ethics Committee of the Center for Health Studies at UVG and was classified as ‘Research not involving human subjects’ (Protocol No. 270–05-2022) and was approved by the Institutional Animal Care and Use committee of Universidad del Valle de Guatemala (CEUCA – UVG) under protocol number I – 2022 (3)A. Additionally, this study was approved by the Texas A&M University’s Institutional Animal Care and Use Committee (IACUC 2022–0001 CA).

### Sampling sites

**Figure 1:**
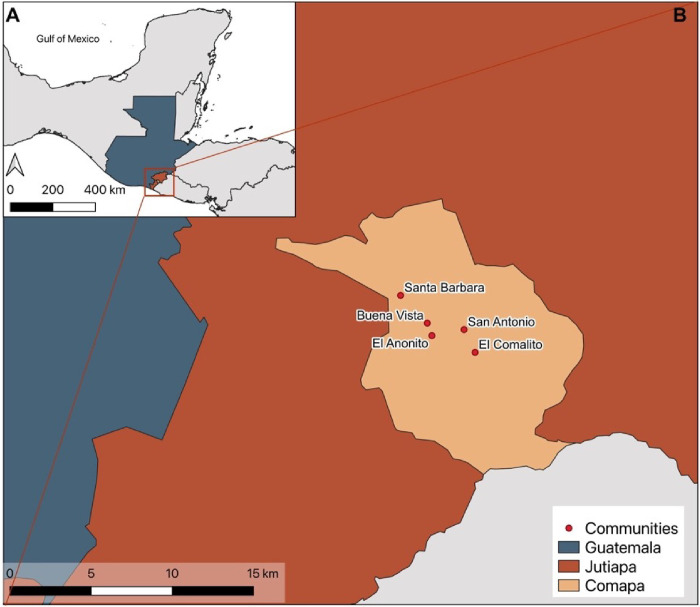
**A.** Map of Guatemala. **B.** Map of the five communities in municipality of Comapa, Department of Jutiapa, in southeastern of Guatemala.

The sampling was conducted in the municipality of Comapa, Department of Jutiapa, in southeastern Guatemala (Fig. 1) from June-August 2022. There are 56 communities in Comapa with a population of > 27,000, of which over 80% live in rural areas and almost 90% live in poverty (11). A total of five communities were randomly selected from a subset of 18 communities from a previous study in the region related to peridomestic animal management for Chagas disease control (11–14). Households surveyed were the same as the ones selected in the previous studies (24 households per community), providing us with information regarding the presence of dogs in the household. Additionally, investigators form UVG have had extensive community engagement activities with these community members. The selected communities were Buena Vista (BV), El Anonito (EA), El Comalito (EC), San Antonio (SA), and Santa Barbara (SB).

### Questionnaire

A questionnaire was designed to survey the house conditions, domiciliary and semi-domiciliary animals, vector presence, pesticide use, and awareness of vector and vector-borne disease. In this study, we focused on the frequency of house animals and ectoparasites in dogs. Therefore, based on the relevance, two variables from the survey were used to analyze their relationship with tick and flea presence and abundance in this study which are 1) Do you use something to protect your dog’s health; 2) Do you use any approaches in your home to prevent or eliminate insects.

### Ectoparasite collection

Dogs owned by the household residents were leashed, muzzled, and restrained with the owners’ permission. Attached ticks were removed using fine-tipped forceps, and a flea comb was used to sample fleas. Households were also inspected for tick infestations by using flashlights to inspect cracks and crevices throughout the home. All collected ectoparasites were immediately stored in 70% ethanol until further examination.

Ectoparasites were morphologically identified to species or genus using taxonomical keys (15, 16). A subset of ticks and fleas were randomly selected and subjected to a molecular identification process (see below).

### DNA extraction and PCRs for arthropod identification and pathogen detection

The DNA of individual ticks and fleas was extracted using the whole body with the exception of eight ticks from which we used only half body and two fleas that were submitted as voucher specimens to the Texas A&M University Entomology Collection (TAMUIC-767). Each ectoparasite was sliced into at least four pieces using a sterile scalpel blade and subjected to DNA extraction using the E.Z.N.A. Tissue DNA Kit (Omega Bio-Tek, GA, USA) following manufacturer’s instructions with overnight lysis (Salomon et al. 2022). A final elution volume of 50 uL was obtained for each sample.

Molecular identification of ectoparasites was performed via PCR with primers targeting the 12S rRNA gene for ticks and the cytochrome c oxidase subunit 1 (COI) gene for ticks and fleas. To amplify a fragment of the 12S rRNA gene, 1.5–3 μL of DNA was used in a 15 μL reaction containing 7.5 uL of FailSafe^™^ 2x PreMix E, 0.25 μL of FailSafe^™^ enzyme (Lucigen, Middleton, WI, USA), 0.5 μL of each primer (5 μM), and molecular grade water and the thermal cycle conditions described in Beati and Keirans (17). Two pairs of COI primers were used to amplify the COI gene: LCO1490 and HCO2198 (18); and LCO1490 and Cff_R (19). With LCO1490 and HCO2198 primers, the reaction consisted of 12.5 μL of FailSafe^™^ 2x PreMix E, 1 μL of each primer (10uM), 0.5 μL of FailSafe^™^ enzyme, 1 μL of DNA sample, and molecular grade water, resulting in a total volume of 25 μL. The thermal cycling condition had an initial denaturation at 94°C for 3 min followed by 35 cycles of 94°C for 30 sec, 50°C for 30 sec, and 72°C for 30 sec, with a final elongation at 72°C for 8 min. Using the LCO1490 and Cff_R primers, reactions of 25 μL contained 12.5 μL of FailSafe^™^ 2X PreMix E, 1 μL of each primer (10 uM), 0.5 μL of FailSafe^™^ enzyme, 2 μL of DNA sample, and molecular grade water. The reaction condition followed the protocol from Lawrence et al. (19).

A quantitative PCR (qPCR) was used to detect the presence of *Rickettsia* species using primers and a probe targeting the citrate synthase protein gene (*gltA*) (20). The reaction consisted of 12.5 μL of iTaq Universal Probes Supermix (Bio-Rad, Hercules, CA), 1.125 μL of each primer (10 uM), 0.375 μL of probe, 5 μL of DNA sample, and PCR water, resulting in a final volume of 25 μL. Positive samples from qPCR were subject to conventional PCR with primers also targeting the *glt*A gene (21). Molecular grade water and a *Rickettsia*-positive tick sample ( (22)) were included as negative and positive controls, respectively, and produced expected outcomes.

A conventional PCR was used to detect *Bartonella* species with primers targeting the *pap31* gene (23). The 25 μL reaction contained 12.5 μL of Premix E, 1.6 μL of each primer (10 μM), 0.25 μL of enzyme, 2.5 μL of DNA template, and PCR water. The reaction was started with a 3 min pre-denature at 95°C, and followed by 44 cycles of 30 sec at 95°C, 30 sec at 58°C, 45 sec at 72°C, then finished with 7 min at 72°C. The DNA from a *B. henselae*-positive flea (24) was used as a positive control and molecular grade water was added as a negative control. All primers used in this study are presented in Table 1.

Amplicons from conventional PCRs were examined using 1% agarose gel electrophoresis, samples producing bands of the expected sizes were purified with ExoSAP-IT (USB Corporation, OH, USA) following the manufacturer’s protocol, and were submitted to bi-directional Sanger sequencing (Eton Biosciences, San Diego, CA). Sequences were examined using UGENE (Unipro LLC, Novosibirsk, Russia) and the consensus was compared to sequences in GenBank using the Basic Local Alignment Search Tool (BLAST) (25). Representative tick, flea, and pathogen sequences were deposited on GenBank (Accession Nos. PP940107–09; PP940828–30; PP952311–14).

### Statistics

Mean intensities of fleas and ticks were calculated by dividing the total number of ectoparasite by the number of infested hosts. Logistic regression was used to explore the effect of four explanatory variables including dog number in the household, dog protection, pesticide uses, and repellent uses, with fleas and tick presence as response variables. When quasi-complete separation occurs, Firth’s bias-reduced logistic regression was used instead. All analyses were conducted using R (version 4.2.2; R Foundation for Statistical Computing, Vienna).

## Results

### Household survey

**Figure 2:**
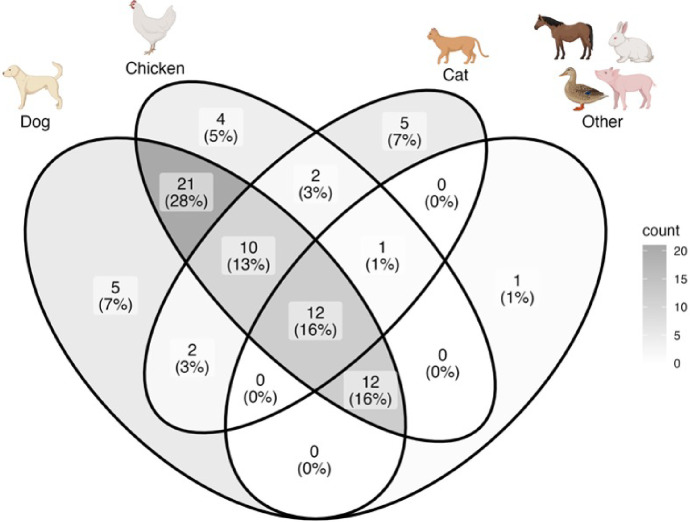
Of 77 households surveyed in Comapa, Jutiapa, Guatemala, in summer 2022, 75 (97.4%) had domestic animals. Houses frequently had multiple species of domestic animals. Values inside boxes represent the number and the proportion (in %) of households owning animals in each of four categories.

Out of 77 households, 75 had domestic animals, including 62 (80.52%) with at least one dog, 32 (41.56%) with at least one cat, and 62 (80.52%) with at least one chicken (Fig. 2). Sixty-two (80.52%) households had more than two species of domestic animals (Fig. 2). On average, there were 1.88 dogs/household (SE = ±0.18) and 0.61 cats/household (SE = ±0.10). Of the households with dogs sampled for ectoparasites, 80% utilized products to protect dogs’ health such as vaccination and shampoo; 62% applied insect management approaches, such as applying pesticides and smoke (Table 2).

### Ectoparasites detected

**Figure 3:**
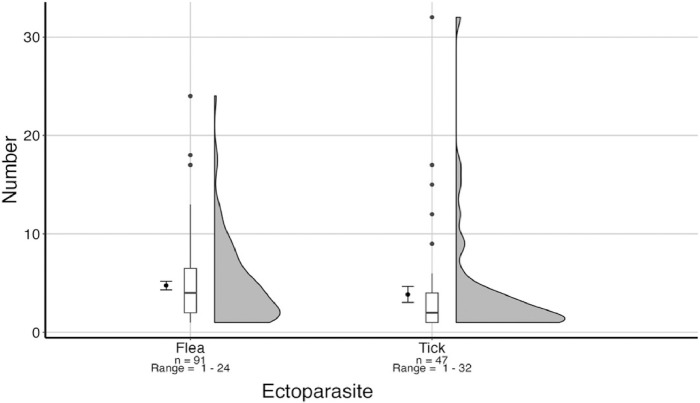
Summary of fleas and ticks collected from infested dogs. The dot and whisker on the left represent the mean intensity and standard error, respectively. The box plots represent the medians, whiskers represent minimum and maximum excluding outliers, which are included as dots. The violin plots to the right represent frequency distribution of ectoparasites collected from dogs in this study.

Of the 133 dogs that were checked for ectoparasites, fleas and ticks were collected from 91 (68.42%) and 47 (35.34%) dogs, respectively, while 27 (19.56%) dogs had both fleas and ticks and 111 dogs (83.46%) had fleas or ticks. From the infested dogs, a total of 433 fleas and 181 ticks were collected with mean intensities of 4.81 (SE = ±0.43) and 3.85 (SE = ±0.80), respectively (Fig. 3). The flea abundance ranged from 0–24 on each dog with a mean abundance of 3.26 (SE = ±0.32), while the abundance of ticks on dogs ranged from 0–32 with a mean abundance of 1.36 (SE = ±0.35). In addition, 33 ticks were collected from house walls.

**Figure 4:**
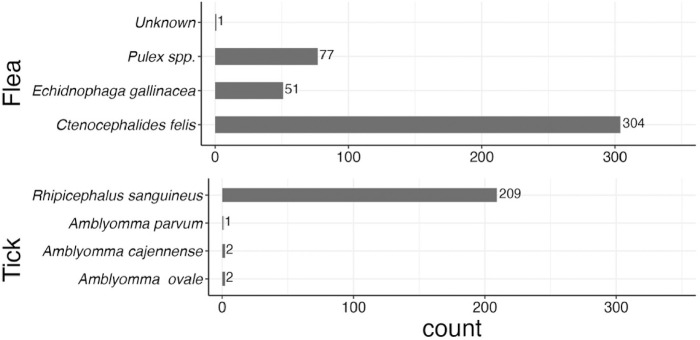
Species and numbers of fleas collected from infested dogs and ticks collected from infested dogs and from house walls in Comapa, Jutiapa, Guatemala, between June and August 2022.

The collected fleas consisted of three species (Fig. 4). While the majority of the fleas (304, 70%) were *Ctenocephalides felis* (cat flea), 51 (11.8%) were *Echidnophaga gallinacea*, the sticktight flea (Fig. 4). There were 77 (17.8%) fleas morphologically identified as *Pulex irritans*, the human flea. DNA sequencing of five of these specimens from three dogs of two households showed 96.6–97.8% identity compared with *P. irritans* (GenBank: MH107045.1) and *P. simulans* (GenBank: OM366056.1), respectively. Because sequences obtained here had an identity of 99.4% with *Pulex* sp. (GenBank: KM891015.1), these fleas are referred to as *Pulex* sp. in this study. Molecular barcoding confirmed the identity of 14 *C. felis*. One flea could not be identified due to extensive damage.

Of the 214 collected ticks (181 ticks from dogs and 33 ticks from house walls), the morphological identifications of 18 (8.4%) of them were molecularly confirmed. Three tick species were identified with almost 98% of them being *Rhipicephalus sanguineus*, and the rest in genus *Amblyomma* including *A. cajennense, A. parvum*, and *A. ovale* (Fig. 4). Exclusively *R. sanguineus* was found on house walls.

### Rickettsia and Bartonella screenings

All ticks (n = 214) and fleas (n = 431), except the two voucher flea specimens (one *C. felis* and one *E. gallinacean*), were screened for *Rickettsia* and *Bartonella* bacteria. Ten samples were positive for *Rickettsia* including seven *C. felis* (2.3%), one *Pulex* sp. (0.5%), and two *R. sanguineus* (1.0%) from dogs in four households (Table 3). The sequences from all positive ticks and fleas matched *R. felis* (100% identity; GenBank: CP000053; (26)) except the sequences from one *C. felis* that matched Candidatus *Rickettsia senegalensis* with 100% identity (GenBank: KF666472; (27)). While no ticks were positive for *Bartonella* sp., 14 fleas were positive including three *Pulex* sp. infected with *B. vinsonii* subsp. *Berkhoffii* (*Bvb*, 100% identity; GenBank: CP003124; (28)), *B. henselae* (100% identity, GenBank: CP072898; (29)), and *Bartonella* sp., respectively. We only obtained a 110 bp fragment deemed as of high quality for the latter sample, precluding us from assigning this sequence to a *Bartonella* species. An additional 11 *C. felis* (3.6%) were infected with *B. henselae*. A dog from EC had seven fleas and two ticks, one of each were positive for *R. felis*, and a dog from SB had four fleas and one tick that were all positive for *R. felis*. Three dogs from SA had multiple fleas (3 out of 4, 6 out of 9, and 3 out of 4) positive to *B. hensela*e. No co-infections were detected in ticks or fleas.

### Associations between vector-control measures and ectoparasite infestation

Three questions from the survey and the number of dogs in households were selected as explanatory variables to evaluate the relationships with ectoparasite infestations. The logistic regression analysis revealed that there is no significant relationship between those variables (Table 4).

## Discussion

This study documents that over 80% of Guatemalan households in five rural communities near the border with El Salvador have at least one dog, 68% of which were infested with fleas and 35% with ticks. *Rickettsia felis* was detected in six *C. felis*, the primary vector that has been found infected globally, including in Guatemala by Troyo, Álvarez (7). These authors reported a high detection rate (54%) in *C. felis* pools collected from the Department of Jutiapa during 2009–2010 (pools can test positive when one or more fleas in the pool is infected; in contrast, we tested fleas individually). Later, the first human case of *R. felis* in Guatemala was reported in a three-year-old boy sampled in 2017 (8). *Rhipicephalus sanguineus* is another species that has been documented to harbor *R. felis* in multiple countries such as Mexico (30), Chile (31), and Brazil (32). However, there are fewer reports of infections of *R. felis* in *Pulex* spp., which prior to our study was only reported in *Pulex irritans* from the Democratic Republic of the Congo (33), Colombia (34), and the United States (35). With multiple vector species on dogs harboring *R. felis*, there could be a risk of infections in humans given the close interactions between humans and dogs. This is important because *C. felis, Pulex* sp. and *R. sanguineus* also feed on humans (36–38), showing the importance of controlling ectoparasites in companion animals as a measure to protect human health.

In addition to *R. felis*, Candidatus *R. senegalensis* was detected from one *C. felis* sample, the first report in Guatemala. Candidatus *R. senegalensis* was first described from *C. felis* collected from Senegal in 2015 (27), and was later detected in Israel (39), Colombia (40), and in California and South Carolina, US (41–43). Although its pathogenicity in humans and other animals remains unclear (44), it has previously been detected in cat tissue (43), and rickettsiae with similar sequences (98.5%) were detected in human blood in Senegal (39).

We detected two *Bartonella* species in fleas removed from dogs in Guatemala. In total, six *C. felis* (1.4%) and one *Pulex* sp. (0.2%) were positive for *B. henselae*, the causative agent of cat scratch disease. Bai et al. (9) reported a higher prevalence (22.4%) of *B. henselae* infection in *C. felis* collected from cats in Guatemala; cats are the primary reservoir for *B. henselae* whereas dogs are less likely to serve as reservoirs for *B. henselae* (45). The detection of *B. vinsonii* subsp. *Berkhoffii* (*Bvb*) in one *Pulex* sp. (0.2%) sample is the first report of *Bvb* in Guatemala. This agent was first isolated from dogs in 1993 (46) and can cause disease in both humans and dogs (47, 48). In surveys conducted in Africa, Asia, South America, the seroprevalence of *Bvb* ranged from 3–65% in dogs (49), which are likely serving as the reservoir of *Bvb* (50). Wild carnivores, such as coyotes (*Canis latrans*), red fox (*Vulpes vulpes*), and raccoon (*Procyon lotor*), may also serve as reservoirs (51, 52) where antibodies to *Bvb* were detected in coyotes with a prevalence of 7–51% across California (53) and 71% in Colorado (51). While the vector for *Bvb* remains unknown, *Pulex* fleas collected from dogs in Florida had been reported to harbor *Bvb* (54), similar results were also documented in Costa Rica (55) and Italy (56). Besides *Pulex* fleas, *Bvb* was also detected in *Ctenocephalides* fleas (57, 58), indicating a wide range of potential vectors for *Bvb* and increasing risk of exposure to *Bvb* from the interaction between humans and domestic dogs.

A total of 77 households were surveyed in this study to obtain knowledge of the domiciliary and semi-domiciliary animals, vector presence, and pesticide use. Most of the households have at least one animal, with dogs and chickens being the most common species in and around the households we visited. Poultry is common across Guatemala, being important to the family as a source of income and nutrition, especially in rural areas (59), while dogs are common in households partially for security reasons. The high populations of animals readily provide blood meal resources for multiple arthropod vectors, including ticks and fleas, in high numbers, increasing the contact risk between vectors and humans. Although 88% of the households that have dogs mention the use of at least one method to protect dogs’ health or manage insects, nearly 68% and 35% of the examined dogs were infested with fleas and ticks, respectively. These results could be attributed to multiple factors such as incorrect application of pesticides (60) and vector resistance development. Therefore, future studies evaluating the ectoparasite management approaches and resistance in the communities should be considered.

## Conclusions

Flea and tick-borne pathogens circulate among dogs and their ectoparasites in rural Guatemala and the knowledge of these transmission cycles can inform the risk of human exposure. Further research is needed on these vector-borne disease threats in neglected regions of Central America.

## Figures and Tables

**Table 1:** Oligonucleotides used in the study for ectoparasite identification and pathogen detection and sequencing.

Gene	Primers	Size	PCR assay	Reference
12S rRNA	T1B F: AAACTAGGATTAGATACCCT	360	Tick identification	(17)
	T2A R: AATGAGAGCGACGGGCGATGT			
COI	LCO1490: GGT CAACAAAT CATAAAGATATTGG	710	Tick and flea identification	(18)
	HCO2198: TAAACTTCAGGGTGACCAAAAAATCA			
	LCO1490: GGT CAACAAAT CATAAAGATATTGG	601	Flea identification	(19)
	Cff-R [S0368]: GAAGGGTCAAAGAATGATGT			
Citrate Synthase	CS-5: GAGAGAAAATTATATATCCAAATGTTGAT	147	*Rickettsia* screening	(20)
	CS-6: AGGGTCTTCGTGCATTTCTT			
	CS-FAM: CATTGTGCCATCCAGCCTACGGT			
	RrCS 372: TTTGTAGCTCTTCTCATCCTATGGC	617	*Rickettsia* screening	(21)
	RrCS 989: CCCAAGTTCCTTTAATACTTCTTTGC			
*Pap31*	PAPn1:TTCTAGGAGTTGAAACCGAT	269	*Bartonella* screening	(23)
	PAPn2: GAAACACCACCAGCAACATA			

**Table 2:** Survey results of the dog health protection and insect management from the house with the dogs sampled for ticks and fleas in Comapa, Jutiapa, Guatemala, in summer 2022.

Question	Response	No. positive responses/Total (%)
Dog health protection	Use dog protection	48/60 (80.0%)
What type of dog protection?	Vaccination	41/60 (67.2%)
	Fumigate	4/60 (6.7%)
	Shampoo	4/60 (6.7%)
	Insecticide	3/60 (5.0%)
	Deworming	2/60 (3.3%)
	Burned oil	1/60 (1.7%)
	Clean nest	2/60 (3.3%)
Insect management	Use some kind of methods to manage insects	37/60 (61.7%)
What types of insect management?	Fumigate	5/60 (8.3%)
	Cleaning	6/60 (10.0%)
	Commercial pesticide (Raid, Folidol, Baygon, Amitraz, Oko, Autan)	23/60 (38.3%)
	Smoke	5/60 (8.3%)
	Electric rackets	2/60 (3.3%)

**Table 3:** Screening results of fleas and ticks removed from dogs and house walls in Comapa, Jutiapa, Guatemala, in June-August 2022, for *Rickettsia* and *Bartonella*

Species	Number of tested	No. infected (%) with *Rickettsia* sp.		No. infected (%) with *Bartonella* sp.		
		*Rickettsia felis*	*Candidatus Rickettsia senegalensis*	*Bartonella vinsonii* subsp. *berkhoffii*	*Bartonella henselae*	*Bartonella* sp.
Flea						
*Ctenocephalides felis*	303	6 (2.0)	1 (0.3)	0	11 (3.6)	0
*Echidnophaga gallinacea*	51	0	0	0	0	0
*Pulex* spp.	76	1 (0.5)	0	1 (1.3)	1(1.3)	1(1.3)
Unknown	1	0	0	0	0	0
Total	431	7(1.6)	1 (0.2)	1 (0.2)	12 (2.8)	1 (0.2)
Tick						
*Rhipicephalus sanguineus*	209	2 (1.0)	0	0	0	0
*Amblyomma ovale*	2	0	0	0	0	0
*Amblyomma cajennense*	2	0	0	0	0	0
*Amblyomma auricularium*	1	0	0	0	0	0
Total	214	2 (0.9)	0	0	0	0

**Table 4:** Logistic regression analysis of potential factors of affection ectoparasite infestation on dogs.

Response variables	Explanatory variables	Levels in model	Odd ratio	95% Confidence interval	*P*-value
Flea presence	Dog number in the household		1.50	0.83–4.69	0.21
	Dog protection	No	Reference		
		Yes	0.73	0.07–4.61	0.75
	Insect management	No	Reference		
		Yes	0.27	0.03–1.41	0.13
Tick presence	Dog number in the household		1.23	0.83–1.91	0.32
	Dog protection	No	Reference		
		Yes	2.48	0.59–12.91	0.23
	Pest management	No	Reference		
		Yes	0.51	0.15–1.61	0.26

## Data Availability

Parasite voucher specimens are submitted to the Texas A&M University Entomology Collection (TAMUIC-767), Gene sequences are available in Genbank with accession nos. PP940107–09; PP940828–30; PP952311–14.
